# A digital image analysis approach to understand the microscopic and macroscopic phenomena in dissolved air flotation

**DOI:** 10.1038/s41598-024-65325-5

**Published:** 2024-06-22

**Authors:** Mohammad Javad Taghizadeh Mohammadi, Salman Movahedirad

**Affiliations:** School of Chemical Engineering, Iran University of Science and Technology (IUST), Tehran, Iran

**Keywords:** DAF, Hydrodynamic, PIV, Flow Pattern, Chemical engineering, Environmental chemistry, Imaging techniques

## Abstract

Dissolved air flotation (DAF) is an effective method for separating suspended oil and solid particles from wastewater by utilizing small air bubbles. This study aims to investigate the impact of key factors, such as saturating pressure and water flow rate, on the separation of fine oil droplets from a water stream. The macroscopic flow patterns within the cell were analyzed using particle image velocimetry (PIV), while Digital Image Analysis (DIA) was employed to study microscopic phenomena, including oil droplet rising velocity and oil-bubble contact mechanisms. Our findings propose a safe operating window (specifically, water flow rate and saturation pressure) for the effective separation of oil droplets without any oil escaping into the clean water stream. It was found that the oil droplet rising velocity increases with the saturation pressure up to 200 kPa. However, a further increase in the pressure of the air saturating chamber leads to a decrease in oil droplet rising velocity. Additionally, we identified a peak in rising velocity at an oil droplet size of approximately 200 µm. Below this threshold, the rising velocity increases with droplet size, while for droplet sizes exceeding 200 µm, the rising velocity decreases with size. This behavior can be explained by the conflicting effects of droplet size increment according to the Stokes law for the rising velocity of oil droplets. As the droplet size increases, the average density of the bubbles/droplet aggregate rises, reducing the ∆ρ in the Stokes law and subsequently lowering the aggregate rising rate. However, as per the Stokes law, the oil droplet rising velocity increases proportionally to the square of its size.

## Introduction

Dissolved air flotation (DAF) treats industrial effluents and clarify water by removing suspended solids, semi-solid particles, and oil droplet^1^. Due to its low retention time, high loading rate, limited space occupation, production of tiny bubbles, and increased interactions of gas bubbles with particles/droplets, this equipment is preferred to the others that rely solely on chemical purification^2,3^. A sudden decrease in the pressure of the air–water mixture stream exiting the air-saturator at a pressure up to 700 kPa results in the formation of microbubbles with sizes ranging from 10 to 100 μm^4,5^. The separation efficiency of equipment such as DAF and induced gas flotation (IGF) is affected by the oil droplet diameter, flow pattern, bubble size, surfactant substances, temperature, and operating pressure. The hydrodynamic design of the DAF equipment, the occurrence of bubbles, and the methods by which oil droplets and gas bubbles are connected result in sufficiently large flocs. As oil/bubbles aggregate has a lower density compared to the water, the formed clots flow to the top more quickly^4,6^.

The impact of hydrodynamic conditions, mass transfer efficiency between the gas and liquid phases, and physical and chemical characteristics of the inlet flow, such as pressure, temperature, pH, etc., can influence the macroscopic and microscopic phenomena involving in a DAF cell^5,7,8^. From a microscopic point of view, bubble–bubble, droplet–droplet, and bubble–droplet interactions should be considered. While from a macroscopic point of view, typically, averaged flow patterns, vortices formed in the cell, and swiping factor are studied.

Using micro-imaging technology, Wang et al.^9^ studied the effect of floc size on the separation efficiency in a flotation cell and indicated that small flocs are more favorable for the high separation efficiency. It was found that the existence of efficient bubble-particle contact is initially controlled by DAF hydrodynamics. Sufficient experimental evidences show that increasing the size of the bubbles reduces the efficient bubble-particle contact^8–10^. However, a reduction in the size of the bubbles should be taken into consideration since tiny bubbles have lower rising velocity and lifting force, which in turn causes more time to rise, and consequently decreases the separation rate^10^.

Structural hydrodynamics, inlet and outlet flow, and the pressure required for saturation were all parameters that altered the flow pattern, improved the oil/bubble contact in the separation region, and hence changed the separation efficiency^1,2^.

In the research study conducted by Montazeri et al.^9^, the impact of adding chemicals on ultrafine nanobubbles stability was examined, but the contact between oil droplets and bubbles in the presence of chemical materials wasn’t determined. They have found that in alkaline solutions, ultrafine bubbles are more stable compared to the bubbles in acidic solutions. Moreover, the presence of low amounts of chloride and sulfate leads to reduced sizes of bubbles as well as at high amounts of these species, the probability of coalescence of bubbles increases.

Moosai et al.^11^ investigated the contact of oil droplets with air bubbles, but it was not presented any real image of the contact of oil droplets and bubbles. According to their outcomes, the impacts of saturation pressure, flow rate, inflow, outflow and hydrodynamic structure have not been studied.

The physical structure and hydrodynamic conditions of the dissolved air flotation equipment were investigated in a recent study by Fanai et al.^5^ Digital image analysis (DIA) is used to detect separation regions and particle interactions with bubbles. The results of their study showed that with changes in the geometry and internal baffles, the overall flow patterns have been changed. The solubility of air in water increases with pressure and causes smaller bubbles and a more homogeneous distribution^12,13^.

Lundh et al.^14^, studied the macroscopic hydrodynamic flow structures in a lab-scale DAF cell using Acoustic Doppler Velocimetry (ADV) technique. Moreover, Terashima et al.^15^, have reported residence time distribution (RTD) of the fluid stream utilizing a tracer. According to their results, changes in the separating baffles in contact and separation regions directly influenced the retention time and separation performance.

Kwon et al.^16^, used computational fluid dynamics (CFD) and analytical fluid dynamics to investigate the impact of the length-to-width ratio of the DAF cell on the hydraulic behavior. Their findings reveal that varying the bubble size has a significant impact on both the flow pattern and dispersion of tiny bubbles. Specifically, when the value is equal to one, it leads to a reduction in the size of the stationary area, resulting in a decrease in the residence time within the cell. Conversely, when the value is equal to five, there is an increase in the dispersion of tiny bubbles, leading to a higher number of collisions between the bubbles and oil droplets, ultimately increasing the residence time within the cell.

While Dissolved Air Flotation (DAF) is a well-established technique for wastewater treatment, it may be less effective than the Induced Gas Flotation (IGF) method in some oily water systems. The bubble sizes generated mechanically in IGF equipment are typically an order of magnitude larger than the bubbles produced through pressure reduction in DAF systems. This disparity could be attributed to various factors related to the relative sizes of bubbles and oil droplets, as well as the reduction in density of the continuous media resulting from homogeneous bubble formation. In this study, we have utilized novel visual techniques such as Digital Image Analysis (DIA) and Particle Image Velocimetry (PIV) to explore micro- and macro-scale phenomena within the contact area of a typical DAF cell. One key observation from this study was the identification of a peak in the oil droplet rising rate relative to its size. Furthermore, a similar trend was noted with an increase in saturator pressure. Our findings revealed that the oil droplet rising velocity increases with saturator pressure up to 200 kPa. However, a further escalation in the pressure within the air saturating chamber results in a notable decrease in oil droplet rising velocity.

## Experimental

### Experimental setup

**Figure 1:**
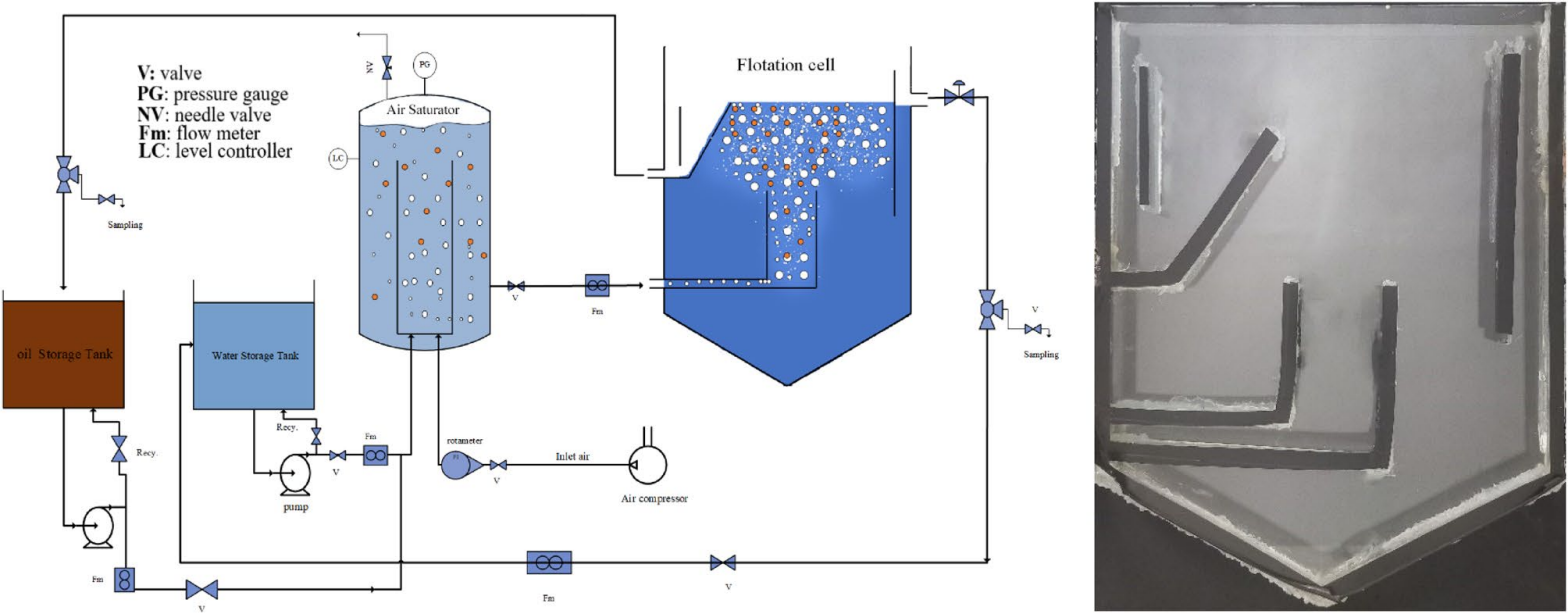
The schematic of the dissolved air flotation (DAF) experimental setup.

The experimental setup considered for this study is demonstrated in Fig. 1. It consists of an air saturation pressure chamber, which is a concentric tube with inner and outer tube diameters of 6 cm and 11 cm, respectively. The height of the inner tube is 45 cm and the outer tube height is 55 cm. Also, an air compressor supplies pressurized air with an output pressure up to 800 kPa. Moreover, the flotation cell was a rectangular glass chamber, as illustrated in Fig. 1. The cell has dimensions of 35 × 45 cm (width × height) while its depth is 2 cm.

The water and pressurized air streams enter the saturation chamber for air saturation at high pressure. Excess air exits from a pressure regulation valve at the top of the saturation chamber, while the saturated water stream flows from the bottom of the annulus toward the flotation cell after passing through a back pressure valve to generate fine bubbles. The flow of air-saturated water was in the range of 1–5 L/min while the pressure of the saturation chamber was set in the range of 100–300 kPa. It should be noted that to capture the rising velocity of oil phase, oil droplet was generated in the range of 50–350 micron by mixing and recirculation of less than 1%w of edible oil in water stream.

A high-resolution camera with a minimum image rate of at least 30 frame per second (fps) at a fixed distance from the flotation cell was used for recording the phenomenon. For image processing, the video was converted into images. Forward light using halogen lamps was utilized to produce high-quality images. The oil droplet size has been measured with image processing technique by using edge detection algorithms of MATLAB software. In addition, the image capturing frame rate was in the range of 30–60 fps at different flow rates. The measurement was carried out in the contact zone of flotation cell.

It should be noted that a black curtain was hung behind the transparent cell and imaging was started after the flow condition was stabilized. Based on the industrial data, a down scaled pseudo-2D flotation cell has been constructed. The clean water was exited from the right-hand side top outlet, while the skimmed oily water was separated from left hand side weir.

### Particle image velocimetry (PIV)

As mentioned previously, the PIV technique was employed to find the flow pattern of gas bubbles inside the flotation cell. Correspondingly, each pair of consequent images was analyzed to obtain the whole domain velocity vector field of the flow stream. In PIV, the cross-correlation function is used to obtain the velocity vector field of the flow stream. Typically, the fluid is seeded with small particles/bubbles. In the present work, the bubbles are presented inherently inside the inlet stream, and it does not need for further particle seeding. The interrogation window size was set to 32 × 32 pixels while an overlap of 50% was selected to obtain more accurate outcomes. Details of the PIV technique utilized in the forward illuminated system can be found in our previous works^17–20^.

## Results and discussion

### Basic observations

In the DAF process, the performance of the separation is affected by physical and chemical conditions, hydrodynamics, the efficiency of mass transfer between the gas and liquid phases, and the size, number, and size distribution of bubbles^2,3^.

**Figure 2:**
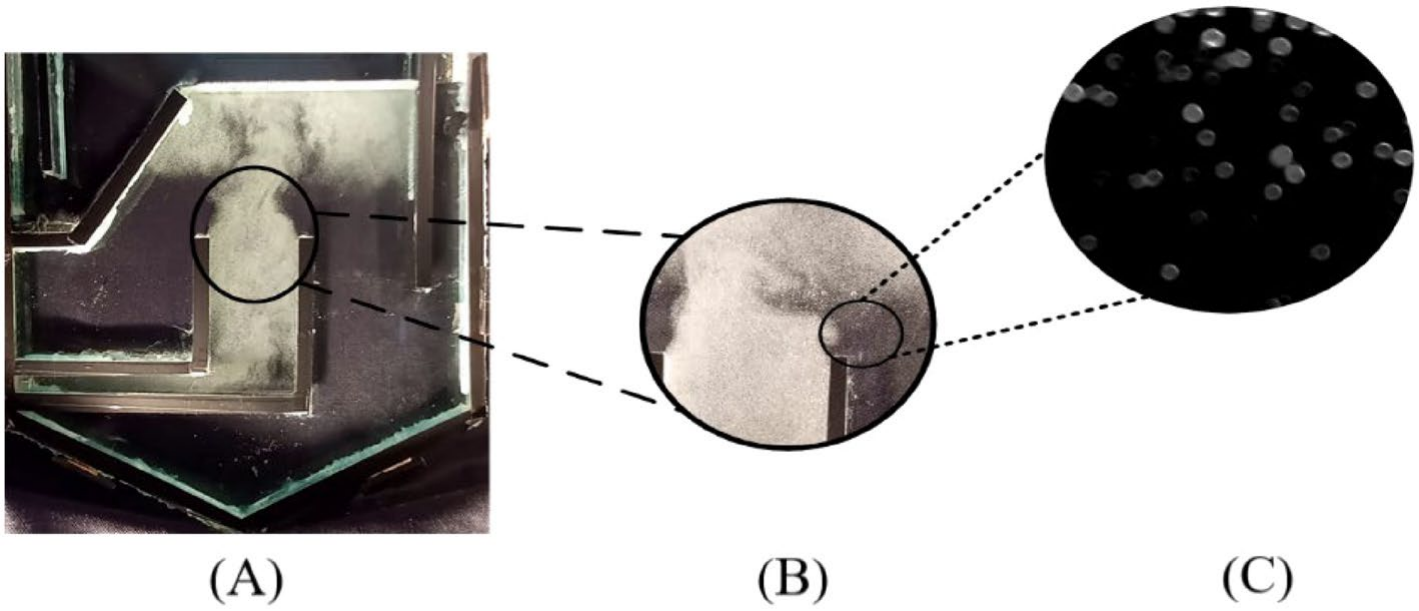
Macroscopic to microscopic phenomena in the DAF cell (**A**–**C**).

The interactions between oil droplets and air bubbles are crucial in various industrial applications, such as oil recovery and foam stabilization. As depicted in Fig. 2, the flow structures can be studied at microscopic and macroscopic scales. The main observations in the microscopic and macroscopic levels are discussed in the following sections.

#### Microscopic phenomena

**Figure 3:**
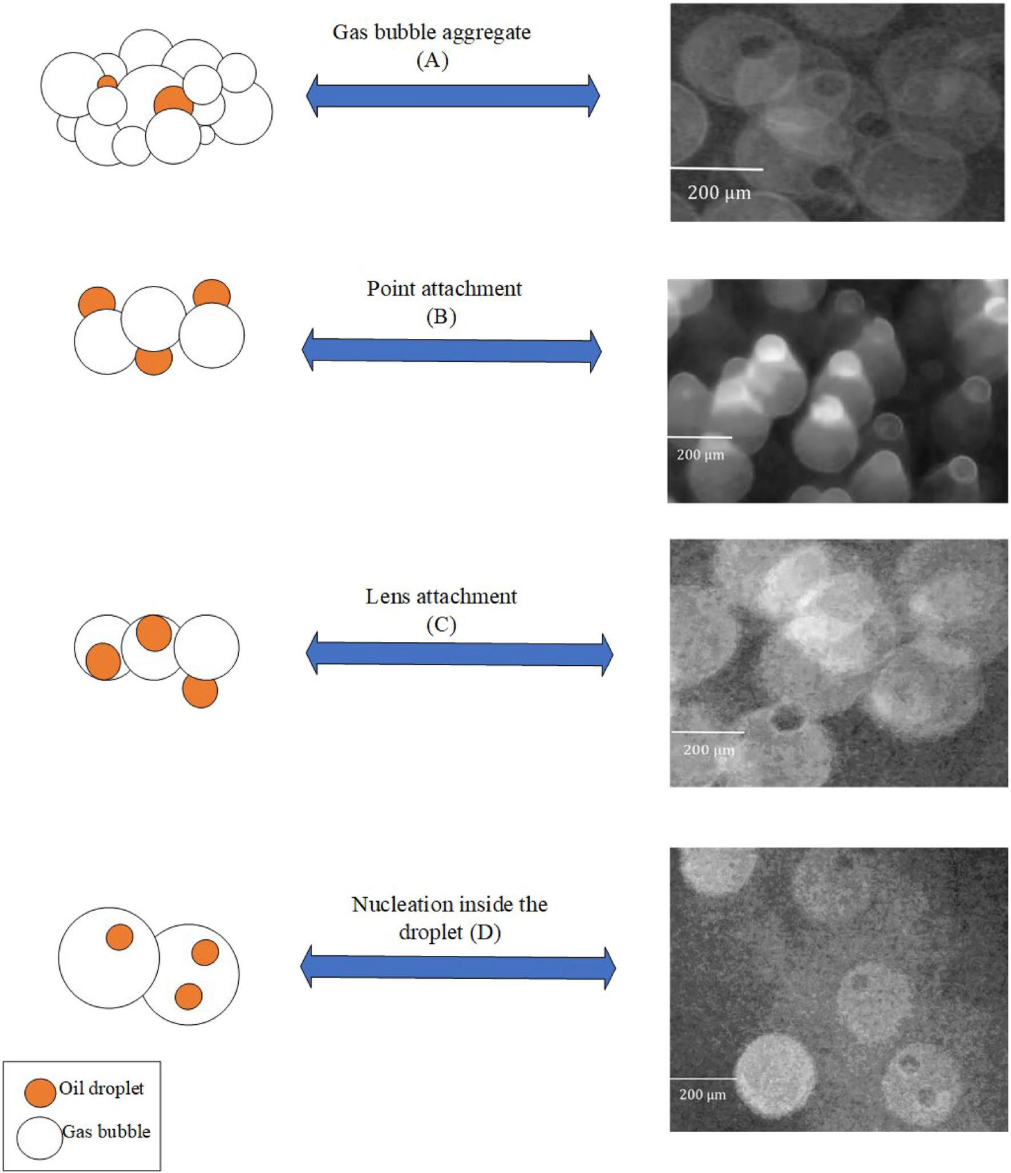
Mechanisms for capturing oil droplets floating in water in gas bubbles: (**A**) gas bubble aggregate, (**B**) point attachment, (**C**) lens attachment, (**D**) nucleation inside the droplet.

From a microscopic point of view, when an oil droplet approaches the air bubbles, the generated bubbles can attach to oil droplets and form aggregates that are both bigger and less dense, as well as thereby easier to remove. Thus, this attachment can occur through different mechanisms, such as formation of an oil film or lens, point attachment, entrapment in a turbulent wake or physical lifting of oil droplets by flocculated gas bubbles (Fig. 3). When the oil droplets are smaller than the air bubble aggregate, the oil droplet is trapped in the bubble aggregate (first mechanism, Fig. 3A).

In Fig. 3B, the point attachment of the air bubble to the oil droplet was observed according to Piccioli et al.^1^, which can be explained by the spreading coefficient equation (${\text{S}}_{0}={\upgamma }_{\text{wg}}-{\upgamma }_{\text{ow}}-{\upgamma }_{\text{og}}$). This equation describes the interfacial tensions of oil with water (${\upgamma }_{\text{ow}}$), air (${\upgamma }_{\text{og}}$), and the water with air (${\upgamma }_{\text{wg}}$), which are critical factors in this type of contact.

Additionally, other mechanisms can occur depending on the values of these interfacial tensions. For instance, if ${\upgamma }_{\text{wg}}<0$ and ${\upgamma }_{\text{ow}}+{\upgamma }_{\text{og}}<0$, the oil does not spread on the surface of the bubble and instead contacts it as a single point (Fig. 3C). On the other hand, when ${\upgamma }_{\text{wg}}<{\upgamma }_{\text{ow}}+{\upgamma }_{\text{og}}$, the contact between the bubble and the oil drop is in the form of a lens (Fig. 3D). Furthermore, smaller oil droplets tend to be trapped inside the air bubbles, causing them to burst and stick together along the flow path. Therefore, understanding the potential contact mechanisms can assist to enhance the performance of oil flotation and achieve better outcomes in industrial processes. Direct contact with the total encapsulation is the ideal mechanism for oil flotation. This produces the strongest bubble/droplet aggregate, which helps avoid separation caused by shear forces inside the flotation cell. Also, when a droplet of oil approaches to the air bubbles, four main processes enter into action, which are illustrated in Fig. 3 at an operating pressure of less than 50 kPa and a discharge flow of less than 1 L/min by placing the camera at the closest distance from the flotation.

#### Macroscopic phenomena

**Figure 4:**
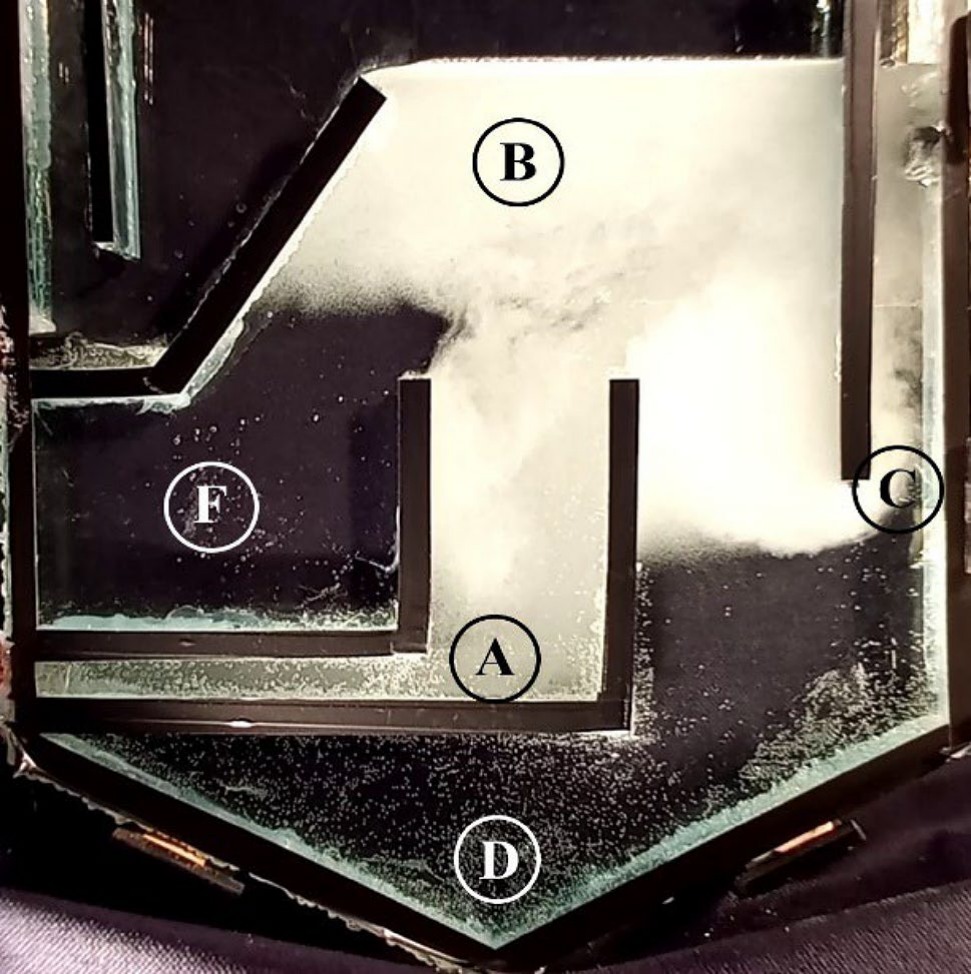
The schematic view of: (**A**) formed bubble, (**B**) contact zone, (**C**) separation zone, and (**D**) sedimentation zones.

When the air-saturated fluid flow passes through the back pressure valve, tiny bubbles are generated in the input pipe, causing the oil droplets to come into contact with the bubbles (Fig. 4, area A). The bubbles’ swarm moves upward, forming macroscopic convective flow structures as large vortices in the contact zone (zone B). In the contact area, the bubbles attach to the oil droplets/small particles and form flocs, which can be separated more quickly compared to the tiny oil droplets. The purified water stream then exits the cell from the effluent zone on the right side of the cell (zone C). If the water stream contains settleable particles, they can be separated at the bottom of the cell (settling zone D).

As can be seen from Fig. 4, there are some dead zones on the left- and right-hand sides of the contact area (zone F). These regions contain fewer bubbles, and the oil droplets/small particles can be trapped in these dead zones.

**Figure 5:**
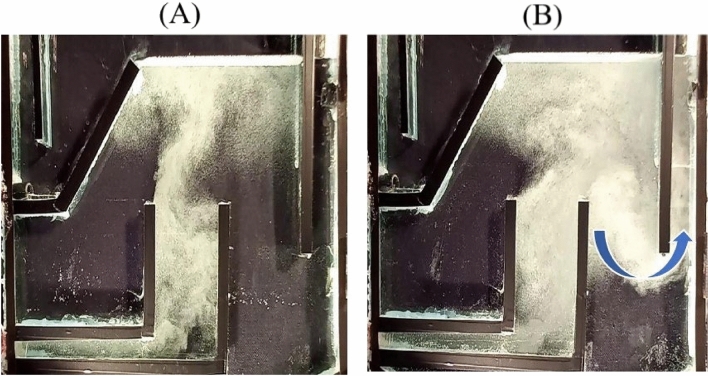
The constant flow rate is 2 L/min: (**A**) saturation pressure of 150 kPa and (**B**) 250 kPa.

Oil droplets adhering to the bubbles can be carried over into the purified water stream if the air saturator pressure or water flow rate is raised, allowing the bubble swarm to exit the effluent zone. The flow structure is shown for two distinct pressures (A and B), with all other parameters being the same in Fig. 5. At low pressure, a diverse pattern is evident, although, in this illustration, the bubbly flow stream escapes partially via the effluent outlet on the right hand side.

**Figure 6:**
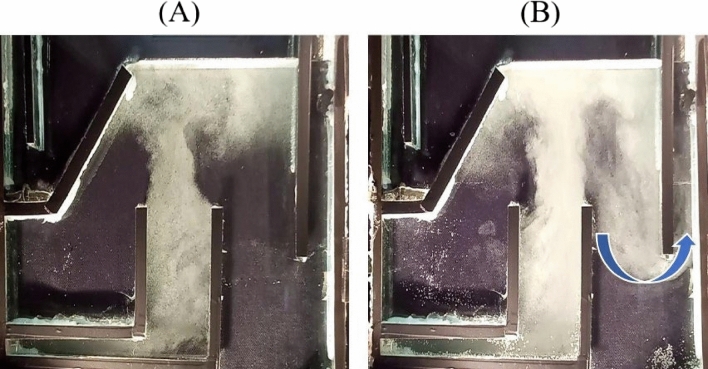
Saturation pressure constant is 200 kPa: (**A**) flow rate 2 and (**B**) 5 L/min.

The same pattern was also observed when, at lower pressures, the flow rate of bubbly streams increased considerably. Figure 6A,B demonstrate the flow stream at an air saturator pressure of 200 kPa with Q = 2 and 5 L/min, respectively. As can be observed, the bubbly flow exits again from the purified water stream in the right-hand side outlet pipe.

**Figure 7:**
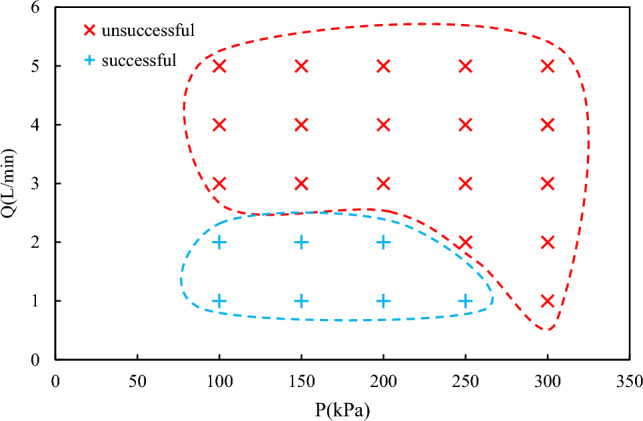
Successful vs. unsuccessful contact scheme map.

**Figure 8:**
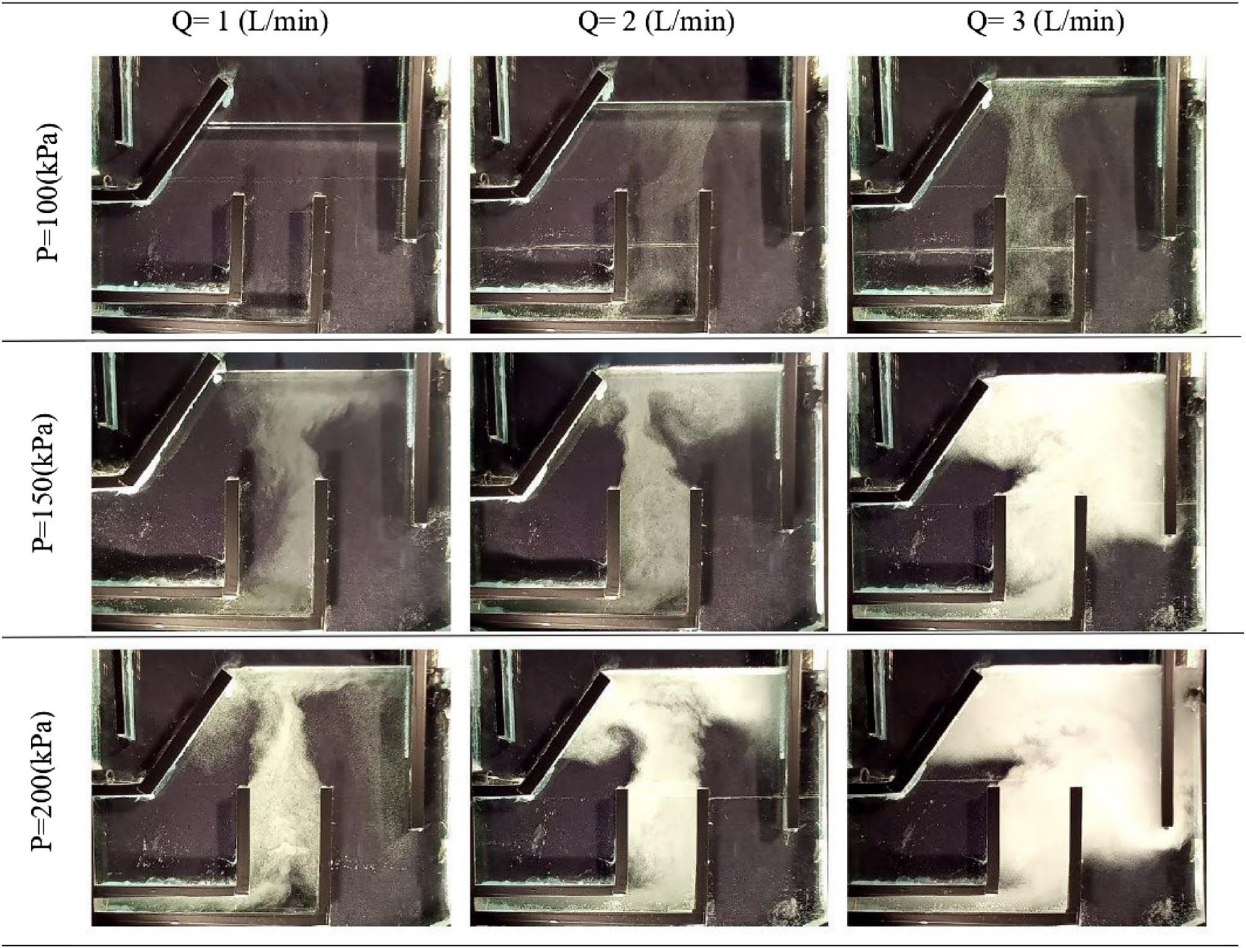
The effect of flow rate and saturation pressure on DAF.

Figure 7 presents the flow map of both successful and unsuccessful separation schemes at varying saturation pressures and water flow rates. The escape of oil-bound bubbles from the treated clean water, as seen in Figs. 5B and 6B, is undesirable and is considered an unsuccessful contact scheme. Therefore, the operational parameters should be adjusted within a safe window to ensure successful oil separation. Figure 8 illustrates the actual conditions of the flotation cell, where the water flow rate and saturation pressure are considered the two main operational parameters. As shown in Fig. 7, the flows in the DAF cell are within the safe operating range when the liquid flow rate and saturation pressures are less than 2 L/min and 250 kPa, respectively. As the pressure in the saturation chamber increases, the number of bubbles generated in the contact cell will also increase. Consequently, the likelihood of a cloudy stream exiting from the right-hand side outlet will increase. Furthermore, an increase in the liquid flow rate inside the cell will result in the formation of two main vortices on both sides of the inlet pipe, as observed in the averaged PIV flow patterns. The size of the vortex loop will increase at higher water flow rates. Therefore, at higher flow rates, the likelihood of a bubbly stream escaping from the right-hand side outlet will also increase.

### Oil droplet rising by gas bubbles

**Figure 9:**
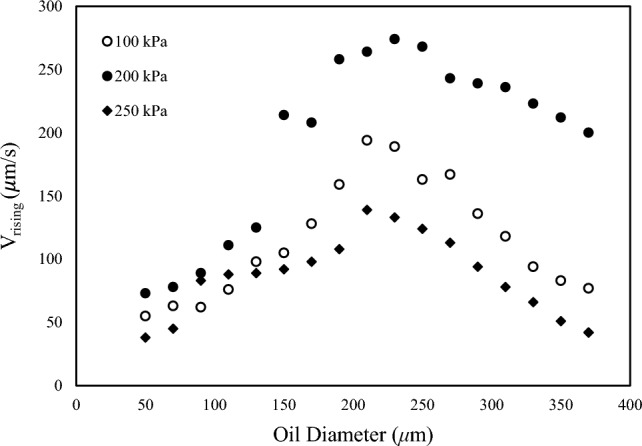
The effect of saturation pressure on the rising velocity rate and particle size at saturation pressures of 100–250 kPa.

**Figure 10:**
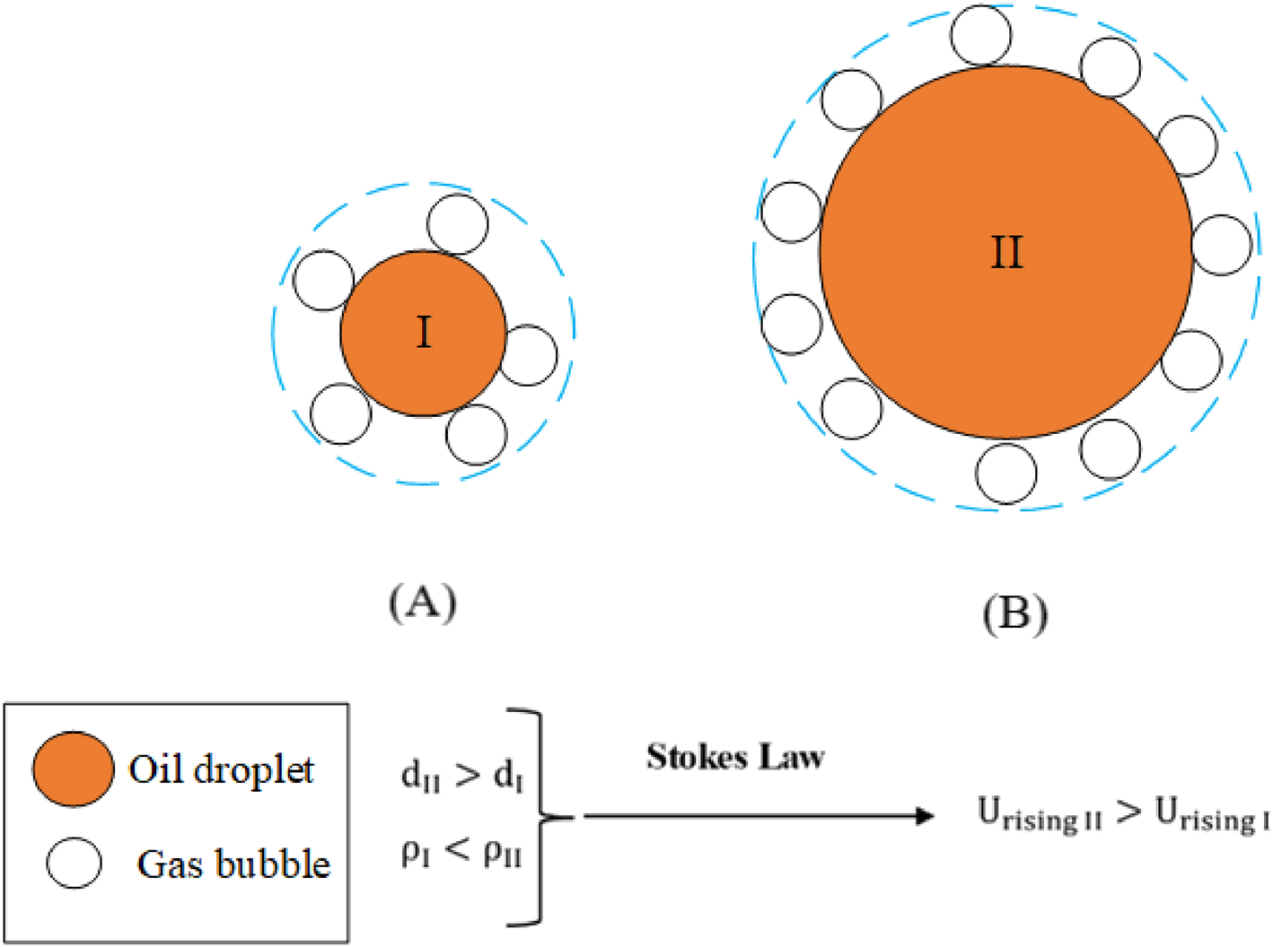
A simplified schematic of the collision of a large and small drop of oil with an air bubble.

As mentioned earlier, gas bubbles enhance the oil droplet rise rate. Indeed, the droplet-bubble combination can increase faster because of the increase in density difference between the bubble and droplet combination and the free stream media. In the present study, the rising rate of oil droplets at different saturation pressures are examined. The results presented in Fig. 9, demonstrate a clear correlation between the saturation pressure and the droplet rising rate. As can be observed in Fig. 9, the rising rate of oil droplets initially increases by increasing the oil droplet size. In contrast, for droplets greater than ~ 200 μm, the trend is different, and the rising rates of droplets decrease by increasing the oil droplet size. The main concept behind this pull–push effect is the Stokes law for the rise of an oil droplet in a quiescent water medium, in which ${\text{U}}_{\text{rising}}\propto$ (${\uprho }_{\text{d}}-{\uprho }_{\text{c}}$)$\times {\text{d}}^{2}$ where ${\uprho }_{\text{d}}$, ${\uprho }_{\text{c}}$ and d refer to discrete and continuous phase densities and oil droplet diameter, respectively. As illustrated in Fig. 10 and according to findings Moosai et al.^17^ when the oil droplet size increases, the density of the oil droplet-small bubbles aggregate (i.e., the discrete phase) tends to the pure oil density. In contrast, at small droplet sizes, the density of the aggregate decreases considerably, and the density difference in the Stokes relation increases. On the other hand, according to Stokes law, by increasing the droplet size, the rising velocity increases. These two contradicting effects lead to an increase in the droplet rising rate at sizes less than 200 μm and a reverse trend at sizes greater than 200 μm.

In other words, a range of gas bubbles is beneficial because the smaller bubbles can capture the smaller oil droplets and the larger ones the larger droplets. Naturally too, the longer the residence time of the gas bubbles in the flotation tanks, the greater the number of gas bubble-oil droplet collisions (contact efficiency), the greater the quantity of the oil that ought to be removed, but clearly water throughput is also an important consideration.

**Figure 11:**
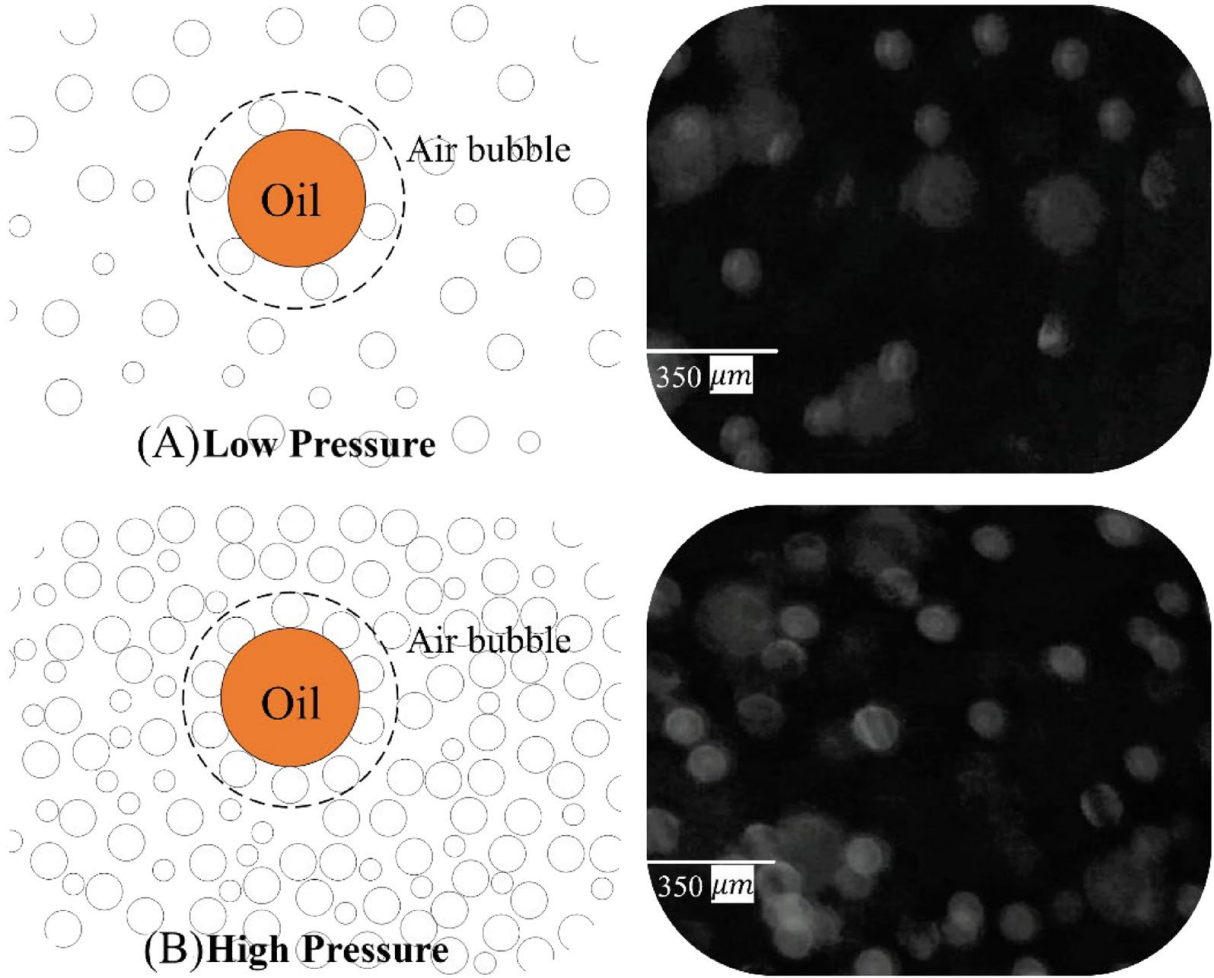
Contact of oil droplets with air bubbles at: (**A**) low and (**B**) high saturation pressures.

Figure 9 also illustrates the effect of saturation pressure on the rising velocity of oil droplet in the flotation cell. As can be observed, up to P = 200 kPa, the rising velocity of droplets increases, while at higher pressures, the rising velocity decreases considerably. This effect can be described again by an analysis of the rising velocity of droplets based on the Stokes equation. According to Stokes law, what is important in oil droplet rise velocity is the difference between continuous phase and droplet phase. The bubbles-droplet aggregate can act as a larger droplet with lower average density. On the other hand, the density in far field (continuous phase) is changed by changing the pressure in the saturation chamber. Figure 11 shows a schematic of two situations at high and low saturating pressures. As can be observed, by increasing the saturation pressure, more fine bubbles will be generated in the DAF cell and consequently the probability of formation of oil/bubbles aggregate will be increased. On the other hand, at higher saturation pressures, more gas is dissolved in the water phase and consequently, more fine bubbles will be released in the DAF cell. Thus, the density of the continuous phase will be decreased, which in turn leads to a decrease in the density difference in the Stokes equation. This pull–push effect is the main concept behind the different trends at P = 250 kPa.

### Particle image velocimetry (PIV)

**Figure 12:**
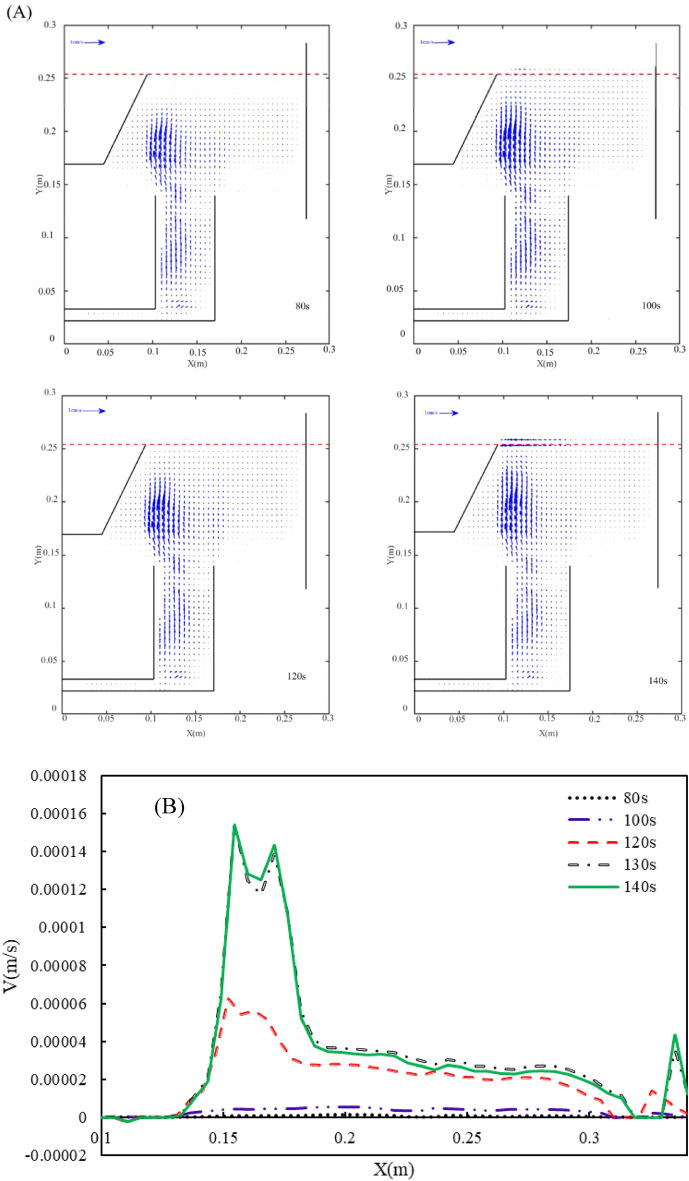
(**A**) The velocity vector field at different times and (**B**) normal velocity profiles at diverse averaging time q = 1 L/min and P = 100 kPa.

**Figure 13:**
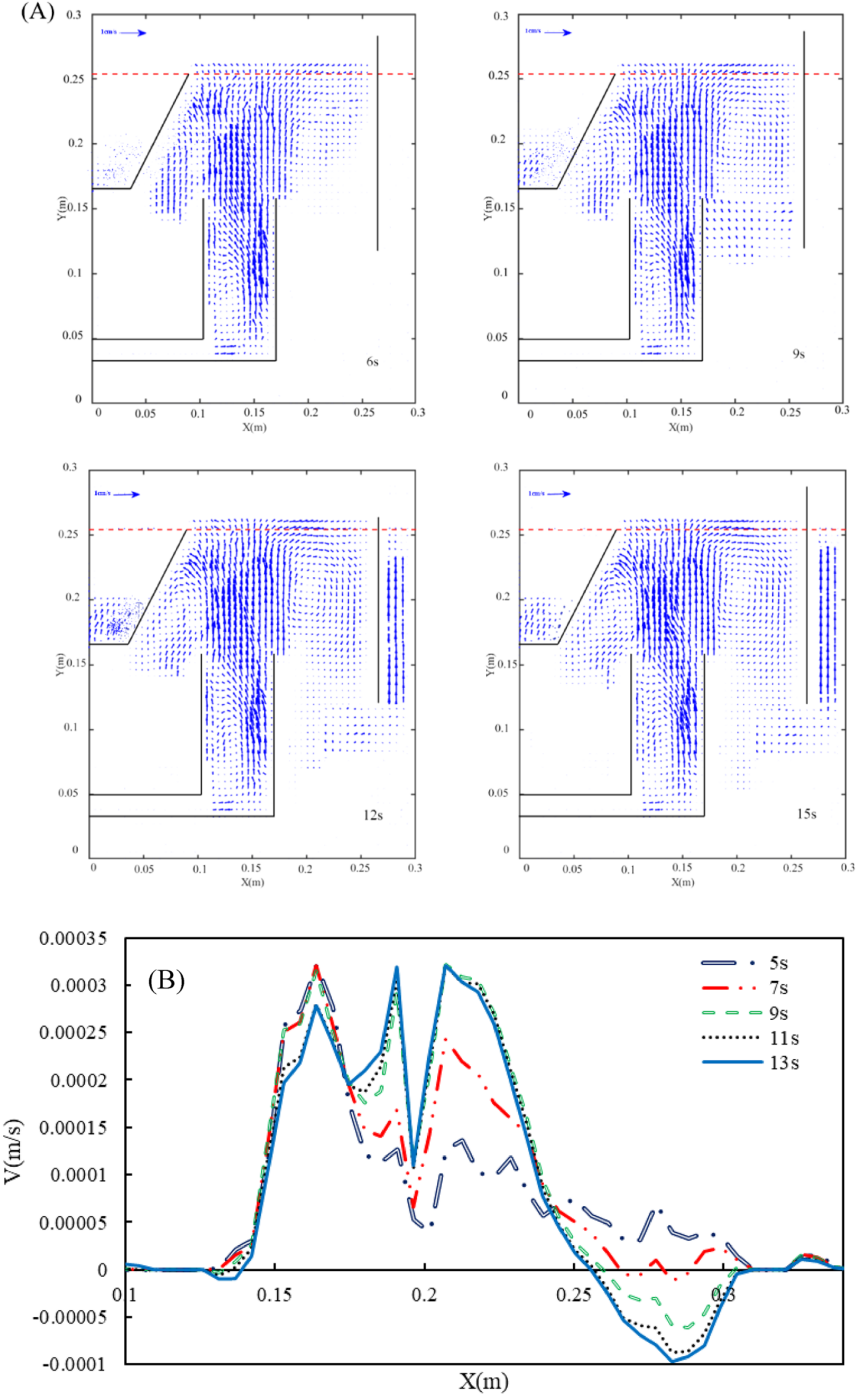
(**A**) The velocity vector field at different and (**B**) normal velocity profiles at different averaging times q = 5 L/min and P = 300 kPa.

PIV technique is employed to find the averaged flow pattern of bubbly flow inside the flotation cell. According to our previous studies^20,21^, in order to make sure about the statistically reliable results, the velocity vector field average over an enough period of time is performed. Thus, the minimum averaging time should be determined for each liquid flow rate. Figure 12 shows the averaged velocity vector field for different averaging times at q = 1 L/min at a saturator pressure of 100 kPa. As can be observed in Fig. 12, the vector field is changed until 120 s, and the trend is the same. This is more precisely illustrated in Fig. 12B, where the average normal velocity at h = 0.25 m from the cell floor is plotted versus x direction distance from the flow inlet stream. This figure shows that after 120 s, the velocity profiles remain unchanged. It should be noted that the minimum averaging time is different for different liquid flow rates. Figure 13 shows the same concept at q = 5 L/min and P = 300 kPa. The figure depicts that for this condition, the minimum averaging time is about 11 s.

**Figure 14:**
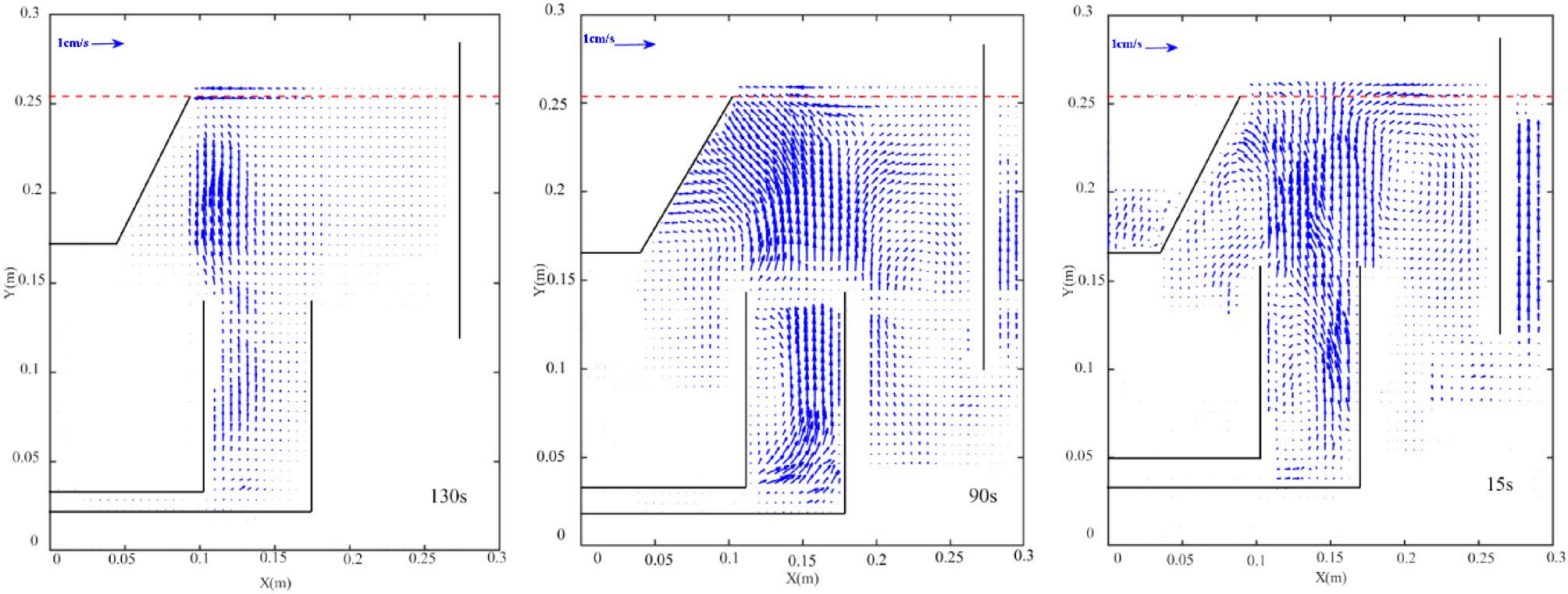
The averaged flow patterns trend at q = 1 L/s and saturation pressures: (**A**) 100 (**B**) 150 (**C**) 300 kPa.

Figure 14 depicts the averaged flow pattern of the gas-liquid mixture at q = 1 L/s but at different saturation pressures (P = 100 kPa to P = 300 kPa). As can be observed by increasing the saturation pressure, two vortexes at the left-top and right-top of the cell are formed. At P = 300 kPa, the volume fraction of gas bubbles increases and the flow contained the gas bubbles exit from the right outlet leg of purified water.

## Conclusion

In the present research study, digital image analysis (DIA) and particle image velocimetry (PIV) techniques are employed to analyze the impact of various factors on DAF design, including flow velocity and saturation pressure. The key findings are as follows:

- Different attachment patterns between the oil droplets and air bubbles were observed, including gas bubble aggregation, point attachment, lens attachment, and bubble nucleation within the droplets.
- The velocity at which oil droplets rise depends on their size and saturation chamber pressure. It was found that at constant saturation pressure, increasing the droplet size leads to a maximum point for rising velocity; afterwards, it tends to decrease. The main concept behind this is the pull–push effect between size and bubbles-droplet aggregate density, according to the Stokes law for oil droplets rising in a quiescent water medium. The size at which the maximum is taking place was about 200 μm.
- On the other hand, for a constant oil droplet size, by increasing the saturation chamber pressure from 100 to 200 kPa, droplet rising velocity increases, while at higher saturation pressure (250 kPa), it tends to decrease. Also, by increasing the saturation pressure, the sweeping effect of bubbles increases, while it has a lightening impact on the continuous phase, which leads to a considerable decrease in the density change between continuous (water bubble mix) and discrete phase (oil droplets).
- The PIV results show two main flow vortexes in the contact zone on the right- and left-hand sides of the cell. At higher saturation pressure and flow rates, it was found that the bubbly flow (which contains oil droplets) can escape from the purified water leg. A flow map for a safe operating window was proposed according to the image processing results.
